# Errata

**DOI:** 10.36660/abc.20230306

**Published:** 2023-05-17

**Arq Bras Cardiol. 2023;120(4):e20230203**

No “Posicionamento Brasileiro sobre Síndrome da Quilomicronemia Familiar – 2023”, com número de DOI: https://doi.org/10.36660/abc.20230203, publicado no periódico Arquivos Brasileiros de Cardiologia, Arq Bras Cardiol. 2023;120(4):e20230203, na página 1, realizar as seguintes correções:

- Incluir o nome da autora Viviane Zorzanelli Rocha Giraldez, cuja instituição é Instituto do Coração (Incor) do Hospital das Clínicas da Faculdade de Medicina da Universidade de São Paulo (HCFMUSP), São Paulo, SP – Brasil, número 7 da lista de instituições.
- Corrigir o nome da autora “Ana Maria Pitta Lottenberg” para “Ana Maria Lottenberg”.

**Edição de Novembro de 2022, vol. 119(5), págs. 815-882**

No “Posicionamento sobre a Saúde Cardiovascular nas Mulheres – 2022”, com número de DOI: https://doi.org/10.36660/abc.20220734, publicado no periódico Arquivos Brasileiros de Cardiologia, Arq Bras Cardiol. 2022; 119(5):815-882, na página 818, realizar a seguinte correção:

Corrigir a declaração de conflito de interesse da autora Maria Cristina de Oliveira Izar:

“Declaração financeira

A - Pagamento de qualquer espécie e desde que economicamente apreciáveis, feitos a (i) você, (ii) ao seu cônjuge/ companheiro ou a qualquer outro membro que resida com você, (iii) a qualquer pessoa jurídica em que qualquer destes seja controlador, sócio, acionista ou participante, de forma direta ou indireta, recebimento por palestras, aulas, atuação como proctor de treinamentos, remunerações, honorários pagos por participações em conselhos consultivos, de investigadores, ou outros comitês, etc. Provenientes da indústria farmacêutica, de órteses, próteses, equipamentos e implantes, brasileiras ou estrangeiras:

- Bayer/Xarelto; Daiichi Sankyo/Lixiana; Libbs/Propafenona e Amiodarona; Pfizer/Eliquis.”

Para:

“Declaração financeira

A - Pagamento de qualquer espécie e desde que economicamente apreciáveis, feitos a (i) você, (ii) ao seu cônjuge/ companheiro ou a qualquer outro membro que resida com você, (iii) a qualquer pessoa jurídica em que qualquer destes seja controlador, sócio, acionista ou participante, de forma direta ou indireta, recebimento por palestras, aulas, atuação como proctor de treinamentos, remunerações, honorários pagos por participações em conselhos consultivos, de investigadores, ou outros comitês, etc. Provenientes da indústria farmacêutica, de órteses, próteses, equipamentos e implantes, brasileiras ou estrangeiras:

- Amgen: Repatha; Amryt Pharma: Lojuxta; AstraZeneca: Dapagliflozina; Aché: Trezor, Trezete; Biolab: Livalo; Abbott: Lipidil; EMS: Rosuvastatina; Eurofarma: Rosuvastatina; Sanofi: Praluent, Zympass, Zympass Eze, Efluelda; Libbs: Plenance, Plenance Eze; Novo Nordisk: Ozempic, Victoza; Servier: Acertamlo, Alertalix; PTCBio: Waylivra.

B - Financiamento de pesquisas sob sua responsabilidade direta/pessoal (direcionado ao departamento ou instituição) provenientes da indústria farmacêutica, de órteses, próteses, equipamentos e implantes, brasileiras ou estrangeiras.

- PTCBio: Waylivra; Amgen: Repatha; Novartis: Inclisiran, Pelacarsen; NovoNordisk: Ziltivekimab.

Outros relacionamentos

Financiamento de atividades de educação médica continuada, incluindo viagens, hospedagens e inscrições para congressos e cursos, provenientes da indústria farmacêutica, de órteses, próteses, equipamentos e implantes, brasileiras ou estrangeiras:

- Novo Nordisk: Diabetes.”

