# Supplementary material for: Errata
Source: Arq Bras Cardiol. 2023 May 17;120(5):e20230306. [Article in Portuguese] doi: 10.36660/abc.20230306 (PMC10348381; doi:10.36660/abc.20230306)
Supplement: Translation

# Translation: Erratum

**Arq Bras Cardiol. 2023;120(4):e20230203**

In the “Brazilian Position Statement for Familial Chylomicronemia Syndrome – 2023”, with DOI: https://doi.org/10.36660/abc.20230203, published in the journal Arquivos Brasileiros de Cardiologia, Arq Bras Cardiol. 2023;120(4):e20230203, on page 1, make the following corrections:

- Include the name of the author Viviane Zorzanelli Rocha Giraldez, whose institution is Instituto do Coração (Incor) of the Hospital das Clínicas of the Faculty of Medicine of the University of São Paulo (HCFMUSP), São Paulo, SP – Brazil, number 7 on the list of institutions.
- Correct the name of the author “Ana Maria Pitta Lottenberg” to “Ana Maria Lottenberg”

**November 2022 issue, vol. 119(5), pages 815-882**

In the “Position Statement on Women’s Cardiovascular Health – 2022”, with DOI: https://doi.org/10.36660/abc.20220734, published in the journal Arquivos Brasileiros de Cardiologia, Arq Bras Cardiol. 2022; 119(5):815-882, on page 818, make the following correction:

Correct the conflict of interest statement of the author Maria Cristina de Oliveira Izar:

“Financial declaration

A - Economically relevant payments of any kind made to (i) you, (ii) your spouse/partner or any other person living with you, (iii) any legal person in which any of these is either a direct or indirect controlling owner, business partner, shareholder or participant; any payments received for lectures, lessons, training instruction, compensation, fees paid for participation in advisory boards, investigative boards or other committees, etc. From the brazilian or international pharmaceutical, orthosis, prosthesis, equipment and implants industry:

- Bayer/Xarelto; Daiichi Sankyo/Lixiana; Libbs/Propafenona e Amiodarona; Pfizer/Eliquis.”

To:

“Financial declaration

A - Economically relevant payments of any kind made to (i) you, (ii) your spouse/partner or any other person living with you, (iii) any legal person in which any of these is either a direct or indirect controlling owner, business partner, shareholder or participant; any payments received for lectures, lessons, training instruction, compensation, fees paid for participation in advisory boards, investigative boards or other committees, etc. From the brazilian or international

pharmaceutical, orthosis, prosthesis, equipment and implants industry:

- Amgen: Repatha; Amryt Pharma: Lojuxta; AstraZeneca: Dapagliflozina; Aché: Trezor, Trezete; Biolab: Livalo; Abbott: Lipidil; EMS: Rosuvastatina; Eurofarma: Rosuvastatina; Sanofi: Praluent, Zympass, Zympass Eze, Efluelda; Libbs: Plenance, Plenance Eze; Novo Nordisk: Ozempic, Victoza; Servier: Acertamlo, Alertalix; PTCBio: Waylivra.

B - Research funding under your direct/personal responsibility (directed to the department or institution) from the Brazilian or international pharmaceutical, orthosis, prosthesis, equipment and implants industry:

- PTCBio: Waylivra; Amgen: Repatha; Novartis: Inclisiran, Pelacarsen; NovoNordisk: Ziltivekimab.

Other relationships

Funding of continuing medical education activities, including travel, accommodation and registration in conferences and courses, from the brazilian or international pharmaceutical, orthosis, prosthesis, equipment and implants industry:

- Novo Nordisk: Diabetes.
